# FNR-Dependent RmpA and RmpA2 Regulation of Capsule Polysaccharide Biosynthesis in *Klebsiella pneumoniae*

**DOI:** 10.3389/fmicb.2019.02436

**Published:** 2019-10-29

**Authors:** Tien-Huang Lin, Chien-Chen Wu, Jong-Tar Kuo, Hsu-Feng Chu, Ding-Yu Lee, Ching-Ting Lin

**Affiliations:** ^1^Department of Urology, Taichung Tzu Chi Hospital, The Buddhist Tzu Chi Medical Foundation, Taichung, Taiwan; ^2^School of Post-Baccalaureate Chinese Medicine, Tzu Chi University, Hualien, Taiwan; ^3^Institute of Biochemistry and Molecular Biology, National Yang-Ming University, Taipei, Taiwan; ^4^Department of Biological Science and Technology, China University of Science and Technology, Taipei, Taiwan; ^5^Biomedical Industry Ph.D. Program, National Yang-Ming University, Taipei, Taiwan; ^6^School of Chinese Medicine, China Medical University, Taichung, Taiwan

**Keywords:** *Klebsiella pneumoniae*, oxygen, FNR, capsule polysaccharide, RmpA, RmpA2

## Abstract

Fumarate nitrate reduction regulator (FNR) is a direct oxygen-responsive transcriptional regulator containing an iron-sulfur (Fe–S) cluster. During anaerobic growth, the [4Fe–4S] cluster in FNR (holo-FNR) binds specifically to DNA, whereas exposure to oxygen results in the loss of its DNA-binding activity via oxidation of the [4Fe–4S] cluster. In this study, we aimed to investigate the role of FNR in regulation of capsular polysaccharide (CPS) biosynthesis, serum resistance, and anti-phagocytosis of *K. pneumoniae*. We found that the CPS amount in *K. pneumoniae* increased in anaerobic conditions, compared to that in aerobic conditions. An *fnr* deletion mutant and a site-directed mutant (*fnr*_3__CA_), with the three cysteines (C20, C23, and C29) replaced with alanines to mimic an FNR lacking the [4Fe-4S] cluster, showed marked increase in CPS amount under anaerobic conditions. A promoter-reporter assay and qRT-PCR confirmed that the transcription of the *cps* genes was repressed by holo-FNR. In addition, we found that holo-FNR could repress the transcription of *rmpA* and *rmpA2*, encoding *cps* transcriptional activators. Deletion of *rmpA* or *rmpA2* in the Δ*fnr* strain reduced CPS biosynthesis, suggesting that RmpA and RmpA2 participated in the holo-FNR–mediated repression of *cps* transcription, thereby regulating the CPS amount, serum resistance, and anti-phagocytosis. Taken together, our results provided evidence that RmpA and RmpA2 participated in the holo-FNR–mediated repression of CPS biosynthesis, and resistance to the host defense in response to oxygen availability.

## Introduction

*Klebsiella pneumoniae* is a gram-negative facultative anaerobe that causes both nosocomial and community-acquired infections, including pneumonia, bacteremia, septicemia, and urinary and respiratory tract infections particularly in patients with underlying diseases (Podschun and Ullmann, 1998). In Asian countries, especially Taiwan and Korea, *K. pneumoniae* is the predominant pathogen responsible for pyogenic liver abscesses in diabetic patients (Han, 1995; Lau et al., 2000; Yang et al., 2009). In recent years, reports of KLA and the spread of hypervirulent strains have increased in western countries (Lederman and Crum, 2005). Furthermore, several *K. pneumoniae* strains producing ESBL and/or AmpC β-lactamase have been widely identified, thereby increasing the difficulty in clinical treatments (Alicino et al., 2015; Ma et al., 2015; Pitout et al., 2015; Lin et al., 2016). Additionally, hypervirulent *K. pneumoniae* strains with carbapenem resistance were reported in China (Gu et al., 2017; Zhan et al., 2017). These strains represent a critical threat for human health.

*Klebsiella pneumoniae*, like many facultative anaerobes in the *Enterobacteriaceae* family, grow under either aerobic or anaerobic conditions (such as the anaerobic environment of the human colon, micro-aerobic environment of different tissues, and the aerobic external environment). Thus, sensing and responding to oxygen availability is essential for the competitiveness of these bacteria and their survival *in vivo*. Oxygen plays a critical role in bacteria–host interaction. In the host innate immune system, oxygen is required for the production of reactive oxygen species and NO for defense against bacterial infections (Green et al., 2014). In many facultative anaerobes, including *Escherichia coli*, *Salmonella enterica*, *Shigella* spp., and *Pseudomonas aeruginosa*., oxygen availability has been reported to modulate the expression of genes involved in metabolic adaption and virulence during infection (Green et al., 2014). However, the effect of oxygen availability on the expression of virulence factors in *K. pneumoniae* remains largely unknown.

Fumarate Nitrate Reduction regulator (FNR) is a direct oxygen-responsive transcriptional regulator in bacteria. It contains an [4Fe–4S] cluster in the N-terminal sensory domain to modulate the C-terminal DNA binding domain in response to oxygen availability. However, the N-terminal sensory domain of FNR contains four cysteine residues (Cys20, Cys23, Cys29, and Cys122) which are required for coordination with the [4Fe–4S] cluster (Kiley and Beinert, 1998). Under anaerobic conditions, the holo-FNR binds specifically to DNA to form a dimer, whereas exposure to oxygen causes oxidation of the [4Fe–4S] cluster, thereby resulting in the loss of its DNA-binding activity (Melville and Gunsalus, 1996). In *E. coli*, a metal-free and apo-FNR was formed under aerobic conditions and was degraded by the ATP-dependent protease ClpXP (Mettert and Kiley, 2005). In many bacterial pathogens, FNR not only regulates anaerobic metabolism but also triggers virulence gene expression during infection, such as those involved in iron transport, toxin production, and type III secretion system (Carpenter and Payne, 2014; Green et al., 2014). However, the regulatory role of FNR in *K. pneumoniae* pathogenesis remains unclear.

Multiple virulence factors, including CPS, lipopolysaccharides, fimbriae, iron-acquisition system, porins, and antibiotic resistance factors, have been identified to be involved in *K. pneumoniae* infection. These virulence factors are processed or embedded in the cell envelope, thus allowing bacteria to internalize nutrients and adhere to diverse surfaces or niches within the human host for successful infection (Wu and Fives-Taylor, 2001; Kawai et al., 2011). Of these virulence factors, CPS is considered the major determinant of *K. pneumoniae* pathogenesis (Sahly et al., 2000; Lin et al., 2004). Acapsular *K. pneumoniae* strains showed less virulence in mouse infection models (Lawlor et al., 2005; Paczosa and Mecsas, 2016). Furthermore, hypervirulent *K. pneumoniae* isolates often carry heavy CPS loads, which could protect the bacteria from phagocytosis and death due to serum factors (Sahly et al., 2000; Lin et al., 2004). The degree of mucoidy has also been positively correlated with successful establishment of infection (Lin et al., 2004; Regueiro et al., 2006). Therefore, stringent control of CPS biosynthesis to encounter the various environmental stimuli is essential for successful *K. pneumonia* infection. Previously, we showed that CPS production was affected by iron availability. The coordination of Fur, the CPS regulators RmpA and RcsA, small RNA RyhB, and iron-sulfur cluster regulator (IscR) for regulating CPS biosynthesis was demonstrated to be a crucial mechanism in response to iron availability (Lin et al., 2010; Huang et al., 2012; Wu et al., 2014). In addition, we also found that environmental glucose stimulated CPS production, which was regulated by cAMP signaling pathway (Lin et al., 2013).

A deeper understanding of *K. pneumoniae* virulence factor expression during infection holds promise for future development of the intervening targets. In this study, we aim to investigate the role of FNR in the regulation of CPS biosynthesis, serum resistance, and anti-phagocytosis of *K. pneumoniae* under anaerobic conditions.

## Materials and Methods

### Bacterial Strains, Plasmids, and Media

Bacterial strains and plasmids and the primers used in this study are listed in Tables 1, 2 respectively. Bacteria were routinely cultured at 37°C in LB medium supplemented with the appropriate antibiotics. The antibiotics used include ampicillin (100 μg/mL), kanamycin (25 μg/mL), and streptomycin (500 μg/mL). The aerobic bacteria were cultured in aerated LB broth with agitation (200 rpm) at 37°C for 16 h. The anaerobic bacteria were statically cultured in LB broth at 37°C in an airtight box, filled with 10% CO_2_ and 90% N_2_ for 16 h.

**TABLE 1:** Bacterial strains and plasmids used in this study.

**Strains or plasmids**	**Descriptions**	**Reference or source**
***K. pneumoniae***		
CG43S3	CG43 Sm^r^	Lai et al., 2001
Δ*fnr*	CG43S3Δ*fnr*	This study
*fnr*_3__CA_	CG43S3*fnr*_3__CA_	This study
Δ*rmpA*	CG43S3Δ*rmpA*	Cheng et al., 2010b
Δ*rmpA2*	CG43S3Δ*rmpA2*	Lai et al., 2003
Δ*fnr*Δ*rmpA*	CG43S3Δ*fnr*Δ*rmpA*	This study
Δ*fnr*Δ*rmpA2*	CG43S3Δ*fnr*Δ*rmpA2*	This study
Δ*lacZ*	CG43S3Δ*lacZ*	Balsalobre et al., 2006
Δ*lacZ*Δ*fnr*	CG43S3Δ*lacZ*Δ*fnr*	This study
Δ*lacZ-fnr*_3__CA_	CG43S3Δ*lacZ-fnr*_3__CA_	This study
Δ*lacZ*Δ*rmpA*	CG43S3Δ*lacZ*Δ*rmpA*	This study
Δ*lacZ*Δ*rmpA2*	CG43S3Δ*lacZ*Δ*rmpA2*	This study
Δ*lacZ*Δ*fnr*Δ*rmpA*	CG43S3Δ*lacZ*Δ*fnr*Δ*rmpA*	This study
Δ*lacZ*Δ*fnr*Δ*rmpA2*	CG43S3Δ*lacZ*Δ*fnr*Δ*rmpA2*	This study
***E. coli***		
BL21(DE3)	*F^–^ ompT hsdS_B_[r_B_^–^m_B_^–^]gal dcm* [DE3]	New England Biolabs
S17-1 *λ pir*	*hsdR recA pro* RP4-2 [Tc:Mu; Km:Tn*7*] [*λpir*]	Miller and Mekalanos, 1988
**Plasmids**		
pKAS46	Ap^r^ Km^r^, positive selection suicide vector, *rpsL*	Skorupski and Taylor, 1996
yT&A	Ap^r^, TA cloning vector	Yeastern
pACYC184	Tc^r^Cm^r^, low copy number cloning vector	New England Biolabs
pfnr	Cm^r^, 1124-bp fragment containing an *fnr* allele cloned into pACYC184	This study
placZ15	Cm^r^, promoter selection vector, *lacZ*^+^	Balsalobre et al., 2006
pOrf12	Cm^r^, 500-bp fragment containing the region upstream of *Klebsiella K2 cps orf1-orf2* cloned into placZ15	Balsalobre et al., 2006
pOrf315	Cm^r^, 900-bp fragment containing the region upstream of *Klebsiella K2 cps orf3-orf15* cloned into placZ15	Balsalobre et al., 2006
pOrf1617	Cm^r^, 300-bp fragment containing the region upstream of *Klebsiella K2 cps orf16-orf17* cloned into placZ15	Balsalobre et al., 2006
prmpAZ15	Cm^r^, 499-bp fragment containing the region upstream of *rmpA* cloned into placZ15	This study
prmpA2Z15	Cm^r^, 615-bp fragment containing the region upstream of *rmpA2* cloned into placZ15	This study

**TABLE 2:** Primers used in this study.

**Primer**	**Sequence (5′→3′)**	**Enzyme cleaved**	
GT305	GGAATTCTGACAATGGATTGCACAA	*Eco*RI	
GT307	TGCAGAGCTCCACCTCTGAGTTATT	*Sac*I	
GT309	GTCGAATTCATCAGCCGTCTGCTG	*Eco*RI	
GT310	GTCTAGATGAGCGGTGGCGGTTAATCG	*Xba*I	
GT311	CGGATCCACGAGGGCTATCTGTTGCTT	*Bam*HI	
GT312	CTGATACTTCGCCATACAGGG		
GT315	GCTAATGCTGGCATCCTGAGCATGGATTG		
GT316	CAGCTCGCCATCCCCTTTACTCTGAACGA		
GT321	CATCCACACCGGGCAAGGGC		
GT322	CCGTCAGCGCGTGGTATCGTGT		
GT411	CGGATCCTGCGCCAGGCATAAAGCTGA	*Bam*HI	
GT412	CAGATCTAATGTAACATCCTTATTGCAC	*Bgl*II	
GT413	CGGATCCCAAGCACCAACTGTTACAC	*Bam*HI	
GT414	GAGATCTGCTTTAGGCCATAATAAAAA	*Bgl*II	

**For**	**Sequence (5′→3′)**	**TaqMan**	**Target**

**qRT-PCR**		**probes**	
RT11	GGTAGGGGAGCGTTCTGTAA	67	23S rRNA
RT12	TCAGCATTCGCACTTCTGAT		
RT17	TCAATAGCAATTAAGCACAAAAGAA	18	*rmpA*
RT18	TTGTACCCTCCCCATTTCC		
RT19	AAATCATTACCCACAACTAACAAAAA	80	*rmpA2*
RT20	TTAGACGGCTTTTTAATTCATGG		

### Construction of *fnr-*Deficient Mutants

Specific *fnr* deletion in *K. pneumoniae* CG43S3 was performed using the allelic exchange strategy described previously (Lai et al., 2003). In brief, the upstream and downstream flanking regions of *fnr* were cloned into the suicide vector pKAS46 (Skorupski and Taylor, 1996), a suicide vector containing *rpsL*, which allows positive selection with streptomycin for vector loss. The resulting plasmid was then mobilized from *E. coli* S17-1λ*pir* (Miller and Mekalanos, 1988) to *K. pneumoniae* CG43S3 or CG43S3-derived strains by conjugation. The transconjugants, with the plasmid integrated into the chromosome via homologous recombination, were selected using M9 agar plates containing ampicillin and kanamycin. Several of the colonies were cultured at 37°C in LB broth supplemented with 500 μg/mL streptomycin to the log phase and then spread onto an LB agar plate containing 500 μg/mL streptomycin. The streptomycin-resistant and kanamycin-sensitive colonies were selected, and the deletion was verified by PCR. The resulting *K. pneumoniae* mutants are listed in Table 1.

### Construction of a *K. pneumoniae fnr_3__CA_* Mutant

A DNA fragment carrying *fnr* and approximately 1000-bp adjacent regions on either side was amplified by PCR using the primer pairs GT321/GT322 (Table 2) and cloned into the yT&A vector. The resulting plasmid was used as the template for inverse PCR with the primer pair GT315/GT316 (Table 2) to generate a mutant *fnr* allele encoding the C20A, C23A, and C29A mutations. The recovered PCR product was treated with *Dpn*I for 2 h, subjected to T4 polynucleotide kinase treatment, and self-ligated with T4 DNA ligase. Subsequently, the mutant allele of *fnr* was subcloned into pKAS46, and the cloning was confirmed by DNA sequencing. Then, the plasmid was mobilized from *E. coli* S17-1 λ*pir* to the *K. pneumoniae* Δ*fnr* strain by conjugation, and the subsequent selection was performed as described above.

### Construction of the pfnr Complementation Plasmid

To obtain the complementation plasmid (pfnr), a DNA fragment containing the promoter and coding sequence of *fnr* was amplified by PCR using the primer pair GT311/GT312 (Table 2) and cloned into the pACYC184 shuttle vector. The ligation product was transformed into *E. coli* DH5α.

### Extraction and Quantification of CPS

CPS was extracted and quantified as previously described (Domenico et al., 1989). The glucuronic acid content, representative of the amount of *K. pneumoniae* K2 CPS, was determined from a standard curve of glucuronic acid (Sigma-Aldrich) and expressed as micrograms per 10^9^ c.f.u. (Blumenkrantz and Asboe-Hansen, 1973).

### Construction of the *rmpA* and *rmpA2* Promoter-Reporter Plasmids

To obtain the promoter-reporter plasmids (prmpAZ15 and prmpA2Z15), the DNA fragments containing the promoter sequence of *rmpA* and *rmpA2* were amplified by PCR using the primer pairs GT411/GT412 and GT413/414 respectively (Table 2), and then subcloned into the placZ15 vector.

### Measurement of Promoter Activity

The promoter-reporter plasmids, pOrf12, pOrf315, pOrf1617, prmpAZ15, and prmpA2Z15-1 were individually transferred into *K. pneumoniae* indicated strains by electroporation. The β-galactosidase activity of bacteria when cultured in LB medium under the indicated condition was measured as previously described (Balsalobre et al., 2006).

### Quantitative Reverse-Transcription PCR (qRT-PCR)

Total RNA was isolated from bacterial cells cultured overnight under anaerobic condition by using the RNeasy midi-column (QIAGEN) according to the manufacturer’s instructions. RNA was treated with RNase-free DNase I (Roche) to eliminate DNA contamination. Then, 100 ng RNA was reverse-transcribed using the Transcriptor First Strand cDNA Synthesis Kit (Roche) with random primers. qRT-PCR was performed in a Roche LightCycler^®^ 1.5 Instrument using LightCycler TaqMan Master (Roche). Primers and probes were designed for selected target sequences using Universal ProbeLibrary Assay Design Center (Roche Applied Science) and are listed in Table 2. Data were analyzed using the real time PCR software of the Roche LightCycler^®^ 1.5 Instrument. Relative gene expressions were quantified using the comparative threshold cycle 2^–ΔΔCT^ method with 23S rRNA as the endogenous reference.

### Bacterial Survival in Serum

Normal human serum, pooled from healthy volunteers, was divided into equal volumes and stored at −70°C before use. Bacterial survival in the serum was determined as previously described (Lai et al., 2003). Briefly, 1 mL of the bacterial cells cultured overnight under anaerobic conditions was washed twice using PBS and resuspended in 1 mL PBS. A mixture containing 250 μL of the cell suspension and 750 μL of pooled human serum was statically incubated at 37°C for 15 min. The number of viable bacteria was then determined by colony counting. The percentage of survival rate was expressed as the number of viable bacteria after incubation with human serum relative to the number of viable bacteria before treatment and then multiplied by 100.

### Determination of the Anti-phagocytosis

The phagocytosis of *K. pneumoniae* strains by RAW264.7 cells was examined as previously described with minor modification (Cheng et al., 2010a). RAW264.7 cells were grown in DMEM (Gibco, Grand Island, NY, United States) containing 10% FBS at 37°C. RAW264.7 cells (4 × 10^5^ cells/well) in 24 well culture plates were co-incubate with anaerobically grown *K. pneumoniae* strains at a ratio of 25:1 (bacteria to RAW264.7 cells). The cell plates were centrifuged at 500 × *g* for 5 min to enhance infection. The cells were incubated for 2 h at 37°C to permit phagocytosis. After 2 h incubation, the cells were washed thrice, then 1 mL of DMEM containing 100 μg/mL of gentamycin were added and incubated for another 2 h to kill the extracellular bacteria. After that, the free bacteria outside the cells were washed with PBS (pH 7.4), and then 0.2 mL of sterile 0.025% Triton X-100 was added to lyse the cells of each well. The mixture in each well was then serially diluted and spread onto LB agar plates for 16 h incubation at 37°C. The plates were incubated at 37°C overnight for colony formation. The number of viable bacteria was then determined by colony counting. The percentage of phagocytosis rate was expressed as the number of viable bacteria incubated with the RAW264.7 cells compared with the number of viable bacteria from the pretreatment and multiplied by 100.

### Statistical Analysis

The experiments for CPS quantification, promoter activity, qRT-PCR analysis, and bacterial survival in serum and phagocytosis were performed in triplicate. The results are presented as the mean and standard deviation. Differences between groups were evaluated by an unpaired *t*-test. Differences with *P*-values < 0.05 and < 0.01 were considered statistically significant.

## Results

### Holo-FNR Represses CPS Biosynthesis Under Anaerobic Growth Condition

**FIGURE 1:**
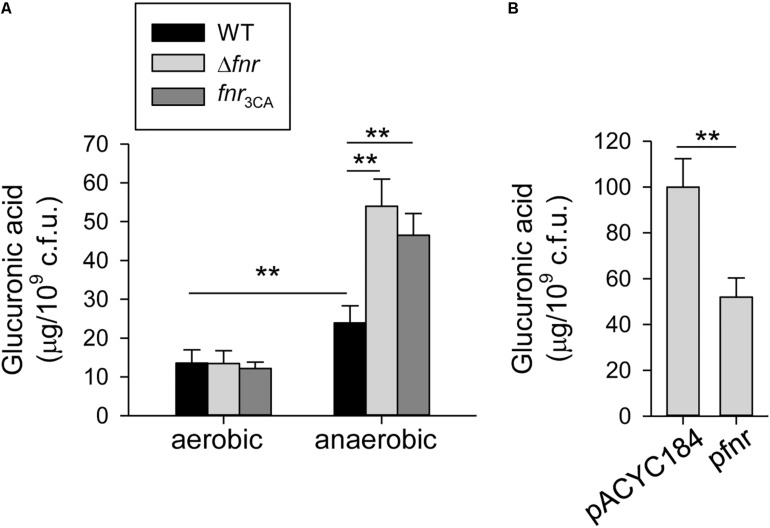
Holo-FNR represses the CPS biosynthesis under anaerobic growth condition. **(A)** CPS amounts of WT, Δ*fnr*, and *fnr*_3CA_ strains grown in LB broth under aerobic and anaerobic conditions were measured. **(B)** CPS amounts of Δ*fnr* carrying pACYC184 or pfnr grown in LB broth under anaerobic condition were determined. After 16 h of growth, the bacterial glucuronic acid content was determined. Error bars indicate standard deviations. ^∗∗^*P* < 0.01 compared to the indicated group.

To observe whether FNR affects the CPS biosynthesis in response to oxygen availability, we determined the amount of CPS in *K. pneumoniae* CG43S3 (WT) and Δ*fnr* strains cultured in aerobic or anaerobic conditions. The CPS amount in anaerobic condition was significantly higher than that in aerobic condition (Figure 1A). Furthermore, *fnr* deletion in *K. pneumoniae* increased the CPS amount, compared to that in the WT strain under the anaerobic growth condition; however, the CPS amount in WT and Δ*fnr* strains was remarkably low under the aerobic growth condition. These results indicate that CPS biosynthesis could be inhibited by FNR in *K. pneumoniae* cultured in anaerobic condition.

In *K. pneumoniae*, FNR contains four highly conserved cysteine residues (C20, C23, C29, and C122 in *E. coli* FNR), which are considered to coordinate the [4Fe–4S] cluster (Kiley and Beinert, 1998). Therefore, to investigate the role of the [4Fe–4S] cluster in FNR regulation of CPS biosynthesis, we created a [4Fe–4S] cluster-deficient *fnr* mutant, *fnr*_3__CA_, by replacing the three cysteines (C20, C23, and C29) with alanines and tested whether this mutant affected CPS biosynthesis. No marked effect was observed in the CPS amount in WT and *fnr*_3__CA_ strains under the aerobic growth condition. However, under anaerobic conditions, the CPS amount in the *fnr*_3__CA_ strain was higher than that in the WT, indicating that the [4Fe–4S] cluster is essential for FNR repression during anaerobic growth. For further complementation analysis, the complete *fnr* were cloned into pACYC184 to yield pfnr. Under anaerobic conditions, the CPS amount in Δ*fnr* [pfnr] was significantly lower than that in Δ*fnr* [pACYC184] (Figure 1B). These results confirmed that FNR in *K. pneumoniae* has a negative role in the regulation of CPS biosynthesis and that the presence of the [4Fe–4S] cluster in FNR is essential for this regulation under anaerobic growth conditions.

### Holo-FNR Acts a Transcriptional Repressor for *cps*

**FIGURE 2:**
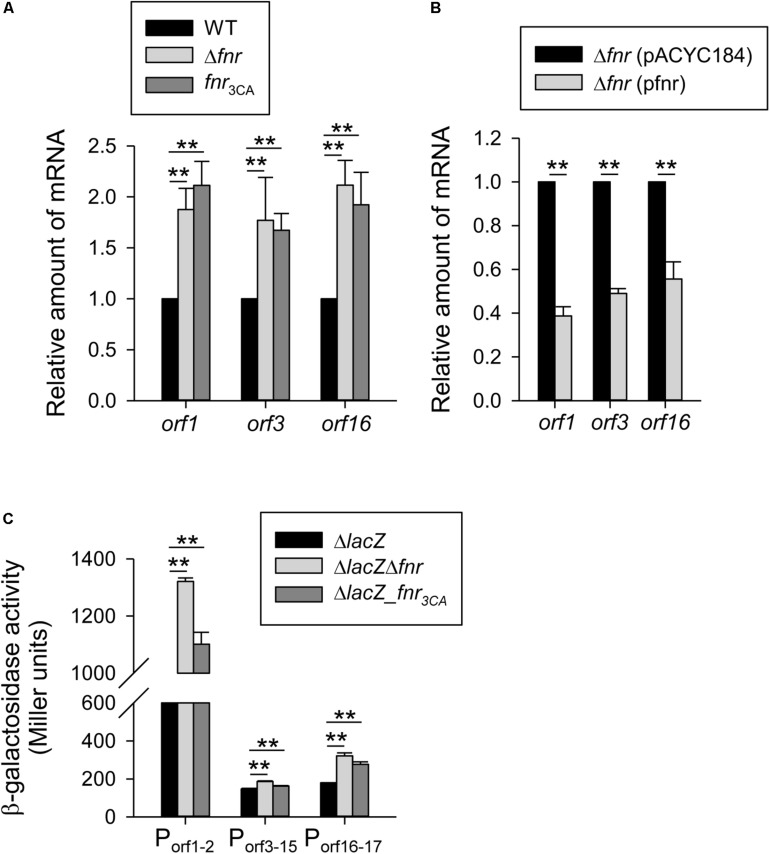
Holo-FNR represses the *cps* transcription under anaerobic growth condition. The mRNA expression of *orf1*, *orf3*, and *orf16* was measured in **(A)** WT, Δ*fnr*, and *fnr*_3CA_ strains and **(B)** the complement strain Δ*fnr* carrying pACYC184 or pfnr which grown in LB medium under anaerobic condition by qRT-PCR analysis. **(C)**β-galactosidase activities of *K. pneumoniae* CG43S3Δ*lacZ* and the isogenic strains (Δ*lacZ*Δ*fnr* and Δ*lacZ*-*fnr*_3CA_) carrying the reporter plasmid pOrf12 (P*_*orf1–2*_*:*lacZ)*, pOrf315 (P*_*orf3–15*_*:*lacZ*), or pOrf1617 (P*_*orf16–17*_*:*lacZ)* were determined using overnight cultures grown in LB medium under anaerobic condition. Error bars indicate standard deviations. ^∗∗^*P* < 0.01 compared to the indicated group.

The K2 *cps* gene cluster of *K. pneumoniae* contains 19 ORFs organized into three transcription units *orf1–2*, *orf3–15*, and *orf16–17* (Arakawa et al., 1995). To investigate the effect of FNR on the expression of the 3 *cps* transcriptional units, the mRNA level of *orf1*, *orf3*, and *orf16* were measured by qRT-PCR in WT, Δ*fnr*, and *fnr*_3__CA_ strains grown in LB medium under anaerobic conditions. As shown in Figure 2A, we found that the mRNA levels of *orf1*, *orf3*, and *orf16* was increased in Δ*fnr*, and *fnr*_3__CA_ strains as compared with the WT. However, the Δ*fnr* strain carrying complement plasmid pfnr could decrease the mRNA levels of *orf1*, *orf3*, and *orf16*, as compared with Δ*fnr* carrying the empty vector (pACYC184) (Figure 2B). In addition, to further observe whether FNR could affect the promoter activity of *cps* gene cluster, we used the reporter plasmids pOrf12 (P*_*orf*__1__–__2_*:*lacZ*), pOrf315 (P*_*orf*__3__–__15_*:*lacZ*), and pOrf1617 (P*_*orf*__16__–__17_*:*lacZ*), each carrying a promoterless *lacZ* gene transcriptionally fused to the putative promoter region of the K2 *cps* gene cluster (Balsalobre et al., 2006), to transform the *K. pneumoniae* strains Δ*lacZ*, Δ*lacZ*Δ*fnr*, and Δ*lacZ-fnr*_3__CA_ strains. Under the anaerobic condition, the promoter activity of *orf1–2*, *orf3–15*, and *orf16–17* in Δ*lacZ*Δ*fnr* and Δ*lacZ-fnr*_3__CA_ strains was higher than that in the Δ*lacZ* strain (Figure 2C). These results indicate that FNR represses the transcription of *cps* genes in an [4Fe–4S] cluster-dependent manner.

To further investigate the mechanism of FNR regulation in *cps* gene transcription, the sequence of the *E. coli* FNR binding site (TTGAT-N4-ATCAA) (Scott et al., 2003) was used to identify the promoter sequence of the K2 *cps* gene cluster for *K. pneumoniae* CG43. Here, the maximum number of possible mismatched nucleotides was set at 2, and only the intergenic regions of the three *cps* transcriptional units were analyzed. Using these criteria, we found that no typical FNR binding site was located upstream of the three *cps* transcriptional units, indicating that the FNR represses *cps* expression indirectly.

### Expression of *rmpA* and *rmpA2* Is Repressed by FNR

Multiple transcriptional regulators have been reported to affect CPS biosynthesis in *K. pneumoniae* CG43, such as CRP, IscR, Fur, RcsA, RcsB, RmpA, RmpA2, KvgA, and KvhR (Balsalobre et al., 2006; Cheng et al., 2010b; Lin et al., 2010, 2013; Wu et al., 2014). Therefore, to further investigate whether these transcription factors are involved in the regulation of *cps* transcription by FNR, the FNR binding site was searched in the upstream sequence of *crp*, *iscR*, *fur*, *rcsA*/*B*, *rmpA*/*A2*, *kvgA*, and *kvhR*. However, we found that the putative FNR binding site is located at −28 to −15 (5′-TTTAT-ATGT-AACAA-3′) and −333 to −320 (5′-TTGTT-TTTA-ATAAA-3′) relative to the translation start site of RmpA and RmpA2, respectively, but no typical FNR binding site was found upstream of the *crp*, *iscR*, *fur*, *rcsA*/*B*, *kvgA*, and *kvhR* sequences. Therefore, these results suggest that RmpA and RmpA2 are involved in the FNR-based regulation of *cps* expression.

**FIGURE 3:**
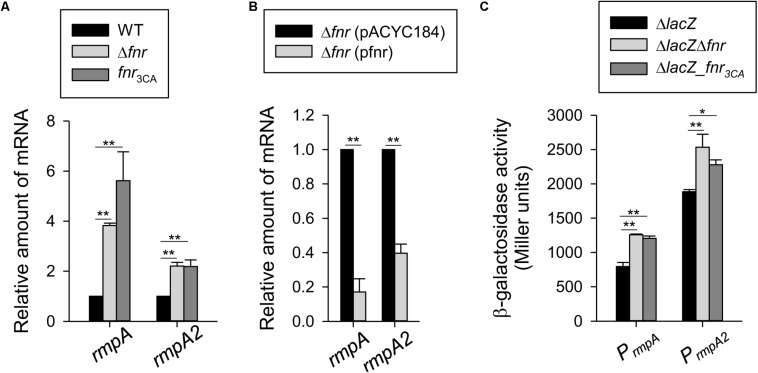
Holo-FNR represses *rmpA* and *rmpA2* transcriptions. **(A)** The mRNA expression of *rmpA* and *rmpA2* was measured in WT, Δ*fnr*, and *fnr*_3CA_ strains and **(B)** the complement strain Δ*fnr* carrying pACYC184 or pfnr which grown in LB medium under anaerobic condition by qRT-PCR analysis. **(C)** The β-galactosidase activities of *K. pneumoniae* CG43S3Δ*lacZ* and the isogenic strains (Δ*lacZ*Δ*fnr* and Δ*lacZ*-*fnr*_3CA_) carrying the reporter plasmids prmpAZ15 (P*_*rmpA*_*:*lacZ*) and prmpA2Z15 (P_rmpA2^*_:*lacZ*) were determined using over-night cultures grown in LB medium under anaerobic condition. Error bars indicate standard deviations. ^∗^*P* < 0.05 and ^∗∗^*P* < 0.01 compared to the indicated group.

To verify this possibility, we first performed qRT-PCR analysis to determine the mRNA levels of *rmpA* and *rmpA2* in the WT, Δ*fnr*, and *fnr*_3__CA_ strains cultured in the anaerobic condition. The mRNA levels of *rmpA* and *rmpA2* in the Δ*fnr* and *fnr*_3__CA_ strains were higher than those in the WT strain (Figure 3A). Furthermore, the Δ*fnr* strain carrying pfnr could decrease the mRNA levels of *rmpA* and *rmpA2*, as compared with Δ*fnr* carrying pACYC184 (Figure 3B). Next, to determine whether FNR functions as a transcriptional regulator for *rmpA* and *rmpA2*, the reporter plasmids prmpAZ15 (P*_*rmpA*_*:*lacZ*) and prmpA2Z15 (P*_*rmpA*__2_*:*lacZ*), each carrying a promoterless *lacZ* gene transcriptionally fused to the putative promoter region of *rmpA* and *rmpA2* respectively, were used to transform the *K. pneumoniae* strains Δ*lacZ*, Δ*lacZ*Δ*fnr*, and Δ*lacZ-fnr*_3__CA_. The promoter activity of *rmpA* and *rmpA2* in the Δ*lacZ*Δ*fnr* and Δ*lacZ-fnr*_3__CA_ strains was higher than that in Δ*lacZ* strain under the anaerobic growth condition (Figure 3C). These results indicate that holo-FNR acts a transcriptional repressor of *rmpA* and *rmpA2* in *K. pneumoniae* during anaerobic growth.

### Role of RmpA and RmpA2 in Regulation of FNR on CPS Biosynthesis

**FIGURE 4:**
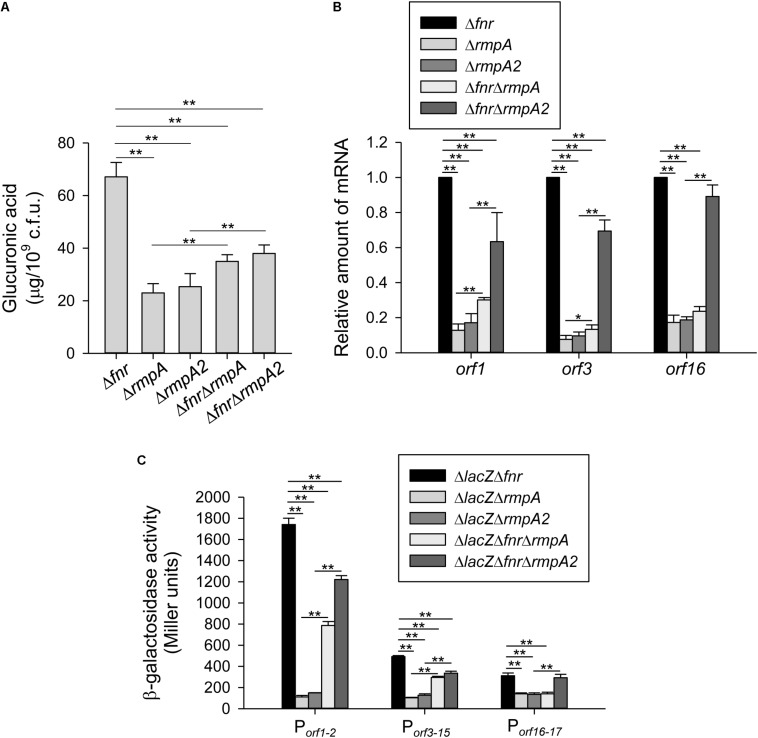
RmpA and RmpA2 are involved in holo-FNR regulation of CPS expression. **(A)** CPS amounts of Δ*fnr*, Δ*rmpA*, Δ*rmpA2*, Δ*fnr*Δ*rmpA*, and Δ*fnr*Δ*rmpA2* strains were determined. Bacterial strains were grown in LB broth under anaerobic condition. After 16 h of growth, the bacterial glucuronic acid content was determined. **(B)** The mRNA expression of *orf1*, *orf3*, and *orf16* was measured in Δ*fnr*, Δ*rmpA*, Δ*rmpA2*, Δ*fnr*Δ*rmpA*, and Δ*fnr*Δ*rmpA2* strains which grown in LB medium under anaerobic condition by qRT-PCR analysis. **(C)** The β-galactosidase activities of *K. pneumoniae* CG43S3Δ*lacZ*Δ*fnr*, Δ*lacZ*Δ*rmpA*, Δ*lacZ*Δ*rmpA2*, and the isogenic strains (Δ*lacZ*Δ*fnr*Δ*rmpA* and Δ*lacZ*Δ*fnr*Δ*rmpA2*) carrying the reporter plasmid pOrf12 (P*_*orf1–2*_*:*lacZ)*, pOrf315 (P*_*orf3–15*_*:*lacZ*), or pOrf1617 (P*_*orf16–17*_*:*lacZ)* were determined using overnight cultures grown in LB medium under anaerobic condition. Error bars indicate standard deviations. ^∗^*P* < 0.05 and ^∗∗^*P* < 0.01 compared to the indicated group.

To investigate whether RmpA and RmpA2 participate in FNR regulation of CPS biosynthesis, the level of CPS was determined in Δ*fnr*, Δ*rmpA*, Δ*rmpA2*, Δ*fnr*Δ*rmpA*, and Δ*fnr*Δ*rmpA2* strains under the anaerobic growth condition. As shown in Figure 4A, the CPS production was reduced in the Δ*fnr* background by the further deletion of *rmpA* or *rmpA2.* However, compared with the Δ*rmpA* or Δ*rmpA2* strains, the CPS production was slightly increased in the Δ*fnr*Δ*rmpA* or Δ*fnr*Δ*rmpA2* strains, respectively. Besides, the qRT-PCR analysis indicated that the mRNA levels of *orf1* and *orf3* were reduced in the Δ*fnr* background by the further deletion of *rmpA* or *rmpA2* (Figure 4B). In addition, compared with the Δ*rmpA* or Δ*rmpA2* strains, the mRNA levels of *orf1* and *orf3* were increased in the Δ*fnr*Δ*rmpA* or Δ*fnr*Δ*rmpA2* strains, respectively. Deletion of *rmpA*, but not *rmpA2*, in the Δ*fnr* strain reduced the mRNA level of *orf16*. The mRNA level of *orf16* was increased in Δ*fnr*Δ*rmpA2* compared with that in Δ*rmpA2*; while no significant difference in the mRNA level of *orf16* was found between the Δ*rmpA* and Δ*fnr*Δ*rmpA* strains. To further validate the regulation of *fnr*, *rmpA*, and *rmpA2* on the expression of *cps* genes, the promoter-reporter assay was performed. As shown in Figure 4C, the promoter activity of *orf1-2* and *orf3-15* were reduced in the Δ*lacZ*Δ*fnr* background by the further deletion of *rmpA* or *rmpA2.* In addition, compared with the Δ*lacZ*Δ*rmpA* or Δ*lacZ*Δ*rmpA2* strains, the promoter activity of *orf1-2* and *orf3-15* were increased in the Δ*lacZ*Δ*fnr*Δ*rmpA* or Δ*lacZ*Δ*fnr*Δ*rmpA2* strains, respectively. Deletion of *rmpA*, but not *rmpA2*, in the Δ*lacZ*Δ*fnr* strain reduced the promoter activity of *orf16-17*. The promoter activity of *orf16-17* was increased in Δ*lacZ*Δ*fnr*Δ*rmpA2* compared with that in Δ*lacZ*Δ*rmpA2*; while no significant difference was found between the Δ*lacZ*Δ*rmpA* and Δ*lacZ*Δ*fnr*Δ*rmpA* strains. These results revealed a complex regulatory circuit in Fnr, RmpA, and RmpA2 that modulate the transcription of *cps* genes in coordination.

### Effect of FNR on Normal Human Serum Resistance and Anti-phagocytosis

**FIGURE 5:**
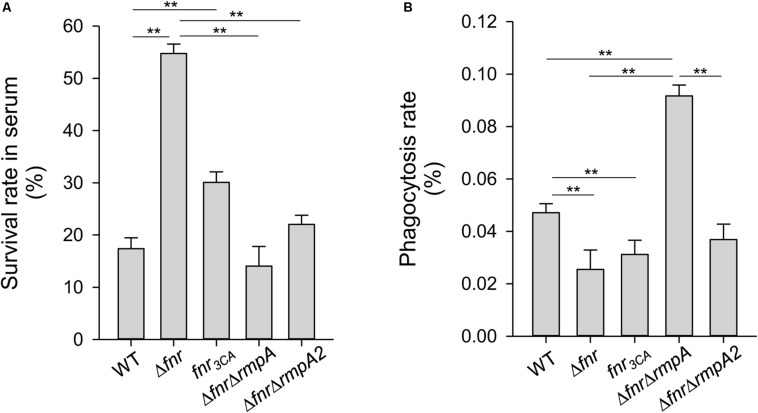
Effects of holo-FNR, RmpA, and RmpA2 on *K. pneumoniae* susceptibility to normal human serum and phagocytosis. The susceptibility to normal human serum **(A)** and the phagocytosis from mouse macrophage RAW264.7 **(B)** of WT, Δ*fnr*, *fnr*_3CA_, Δ*fnr*Δ*rmpA*, and Δ*fnr*Δ*rmpA2* strains which overnight grown in LB medium under anaerobic condition were determined. The survival rate in serum and the phagocytosis rate were quantified as described in section “Materials and Methods.” ^∗∗^*P* < 0.01 compared to the indicated group.

As CPS has been demonstrated to protect *K. pneumoniae* from serum killing and phagocytosis (Sahly et al., 2000; Lin et al., 2004), FNR may also affect the ability of *K. pneumoniae* to resist the bactericidal effects of serum and phagocytosis by regulating CPS levels. To test this hypothesis, we determined the survival rate of anaerobically cultured *K. pneumoniae* strains in 75% normal human serum. Compared with the WT strain, the Δ*fnr* and *fnr*_3__CA_ strains showed a remarkably higher survival rate (Figure 5A), implying the negative role of holo-FNR in the serum resistance of *K. pneumoniae*. To further investigate whether RmpA and RmpA2 are involved in role of FNR in serum resistance, the survival rates of the Δ*fnr*Δ*rmpA* and Δ*fnr*Δ*rmpA2* strains were observed. The deletion of *rmpA* or *rmpA2* in Δ*fnr* strain reduced the survival rate, compared to that in the Δ*fnr* strain (Figure 5A). This confirms the involvement of RmpA and RmpA2 in FNR-mediated regulation of CPS biosynthesis to influence *K. pneumoniae* resistance to normal human serum. Next, to investigate the role of FNR in anti-phagocytosis, the survival rates of the WT, Δ*fnr*, and *fnr*_3__CA_ strains were determined. Deletion of *fnr* and *fnr*_3__CA_ resulted in a marked reduction in the phagocytosis rate, compared to that in the WT strain (Figure 5B). In addition, we also evaluated the phagocytosis rate after the deletion of *rmpA* and *rmpA2* in Δ*fnr* strains. The phagocytosis rate in Δ*fnr* strains lacking *rmpA* was higher than that in the WT and Δ*fnr* strains (Figure 5B). However, the deletion of *rmpA2* in Δ*fnr* strain could restore the effect of phagocytosis to that observed in the WT strain. Collectively, these findings suggest that RmpA and RmpA2 are involved in the negative role of FNR in anti-phagocytosis in *K. pneumoniae* and that RmpA plays a critical role in anti-phagocytosis of *K. pneumoniae*.

## Discussion

Stringent regulation of CPS amounts in *K. pneumoniae* is complex and critical for adaptation to the dynamic environmental signals and for successful infection (Lin et al., 2010, 2013; Huang et al., 2012; Ares et al., 2016; Dorman et al., 2018). Here, we demonstrated that holo-FNR acts a repressor of CPS biosynthesis, thereby influencing *K. pneumoniae* resistance to serum and phagocytosis under anaerobic condition. Furthermore, the involvement of RmpA and RmpA2 in the FNR regulon was also elucidated.

In extraintestinal pathogenic *E. coli* XM, under low oxygen availability, the biosynthesis of extracytoplasmic polysaccharides increased in 100% serum, as compared to that in LB broth (Ma et al., 2018). Similarly, we found that the CPS amounts of *K. pneumoniae* were increased in response to anaerobic growth conditions. However, in this context FNR plays an opposite role, by directly activating the biosynthesis of the K-capsule and colanic acid in *E. coli* (Ma et al., 2018) but repressing the K2 CPS biosynthesis in *K. pneumoniae*. Since the K2 *cps* gene clusters (Arakawa et al., 1995) are relatively different from the genes responsible for the biosynthesis of the K-capsule and colanic acid (Ma et al., 2018), it is reasonable that the regulation of CPS biosynthesis differs between the two bacteria. In addition to FNR, ArcA is a well-studied transcriptional regulator in several bacteria and is known to affect the expression of numerous genes in response to oxygen availability (Green and Paget, 2004). During anaerobic growth, ArcB, a membrane-bound sensor, is auto-phosphorylated and the phosphoryl group is transferred to ArcA, a response regulator, to activate or repress the target gene transcription (Green and Paget, 2004). In *K. pneumoniae*, ArcA promotes persistent colonization in the mouse gastrointestinal tract (Boll et al., 2012); however, the role of ArcBA in *K. pneumoniae* pathogenesis remains unclear. To investigate whether ArcA is involved in regulation of CPS biosynthesis in *K. pneumoniae*, the typical ArcA∼P binding site of *E. coli* (Liu and De Wulf, 2004) was used to analyze the sequences upstream of the three *cps* transcriptional units in *K. pneumoniae*. However, no typical ArcA∼P binding site was found in these sequences. Under anaerobic conditions, ArcA-dependent repression of *fur* transcription has been demonstrated in *E. coli* and *Shigella flexneri* (Liu and De Wulf, 2004; Boulette and Payne, 2007). In *K. pneumoniae*, we found that Fur directly represses the expression of *rmpA*, *rmpA2*, and *rcsA*, subsequently repressing CPS biosynthesis (Lin et al., 2010). Therefore, ArcA may inhibit the Fur-mediated repression of CPS biosynthesis in *K. pneumoniae* under anaerobic growth conditions. However, further studies are warranted to confirm this.

Several studies have reported RmpA and RmpA2 as important virulence determinants for the mucoid phenotype of *K. pneumoniae* (Nassif et al., 1989; Arakawa et al., 1991) In *K. pneumoniae* CG43, deletion of either *rmpA* or *rmpA2* resulted in a marked decreased in *cps* transcription, thereby repressing the mucoid phenotype (Lai et al., 2003; Cheng et al., 2010b). Consistent with this, we found that deletion of either *rmpA* or *rmpA2* in the Δ*fnr* strain showed a remarkable reduction in the CPS amount as compared to that in Δ*fnr* strain. This indicates that RmpA and RmpA2 act as important activators in the FNR-mediated regulation of CPS biosynthesis during anaerobic growth. Under aerobic conditions, RmpA activates the promoter activity of *orf1-2* and *orf16-17* in LB medium, whereas RmpA2 only activates the promoter activity of *orf1-2* (Cheng et al., 2010b). Nevertheless, in anaerobic conditions, we found that the *rmpA*-deletion in the Δ*fnr* strain decreased the promoter activity of the three *cps* transcriptional units; however, the *rmpA2*-deletion in the Δ*fnr* strain reduced the promoter activity of *orf1-2* and *orf3-15* but not *orf16-17.* We considered that the expression of these *cps* gene clusters were differentially regulated by FNR and many other CPS regulators including RmpA/A2, RcsAB, Fur, IscR, and CRP in response to various environmental stimuli (Lai et al., 2003; Cheng et al., 2010b; Lin et al., 2010, 2013; Wu et al., 2014), which may affect the composition, transportation, and assembly of CPS. The regulatory effect of RmpA and RmpA2 on *cps* expression in *K. pneumoniae* cultured in LB medium under anaerobic conditions was similar to that previously observed in *K. pneumoniae* cultured in M9-glucose minimal medium under aerobic conditions (Cheng et al., 2010b). Oxygen and glucose stimuli are considered to affect global protein acetylation in bacteria (Chohnan et al., 1998; Schilling et al., 2015). In addition, protein acetylation is an abundant post-translational modification in bacteria to control protein structure, stability, and function (Wolfe, 2016; Carabetta and Cristea, 2017). Therefore, whether RmpA and RmpA2 could be acetylated to affect its regulatory activity on *cps* expression awaits to be investigated.

In *E. coli*, FNR and CRP bind to a similar DNA sequence, suggesting that the FNR-regulated targets are overlapped to the CRP regulon in anaerobic conditions (Shaw et al., 1983). In *K. pneumoniae*, CRP also acts a transcriptional repressor for the three *cps* transcriptional units (Lin et al., 2013). However, CRP could directly bind to the promoter region of *orf3-15* and *orf16-17* to inhibit the transcription, but CRP-based repression of *orf1-2* transcription is required for inhibiting the *rcsA* expression (Lin et al., 2013). Therefore, although both FNR and CRP repressed the promoter activity of the three *cps* transcriptional units, their regulatory mechanisms are different.

In heavy encapsulated *K. pneumoniae* strains, type 3 fimbriae has been demonstrated to play a crucial role in the biofilm formation both on biotic and abiotic surfaces (Di Martino et al., 2003; Jagnow and Clegg, 2003; Wu et al., 2012). However, the thick capsule of *K. pneumoniae* impedes the assembly and adherence of type 3 fimbriae (Schembri et al., 2005). Thus, tightly controlling the biosynthesis of CPS and fimbriae is critical for successful infection by *K. pneumoniae*. We consider that FNR cross regulates the biosynthesis of CPS and fimbriae in response to oxygen availability during infection, which awaits to be investigated. In addition, we found that holo-FNR repressed serum resistance in *K. pneumoniae* under anaerobic conditions. Deletion of *rmpA* or *rmpA2* in the Δ*fnr* strain restored the survival rate in serum to that observed in the WT strain, indicating the complementary functions of RmpA and RmpA2 in serum resistance. Apart from CPS, LPS in *K. pneumoniae* was also a major determinant in serum resistance in both serotype K1 and K2 strains (Yeh et al., 2016). However, RmpA and RmpA2 have yet been reported to be involved in LPS biosynthesis. Furthermore, we also observed a higher phagocytosis rate in the Δ*fnr*Δ*rmpA* strain, compared to that in the WT and Δ*fnr*Δ*rmpA2* strains. This indicates that compared to RmpA2, RmpA serves a more important role in anti-phagocytosis of *K. pneumoniae*.

In this study, we found that CPS biosynthesis in *K. pneumoniae* is affected by oxygen availability. However, holo-FNR acts an important repressor for CPS biosynthesis, subsequently affecting the ability of serum resistance and anti-phagocytosis via inhibition of *rmpA* and *rmpA2* transcription in *K. pneumoniae* during anaerobic growth. Thus, we demonstrate that FNR plays a critical role in mediating the virulence factor expression in *K. pneumoniae* pathogenesis in response to oxygen availability.

## Data Availability Statement

All datasets generated for this study are included in the manuscript/supplementary files.

## Ethics Statement

For isolation of normal human serum from healthy volunteers, the procedure and the respective consent documents were approved by the Ethics Committee of the China Medical University Hospital, Taichung, Taiwan. All healthy volunteers provided written informed consent.

## Author Contributions

T-HL, J-TK, C-CW, and C-TL conceived and designed the experiments. J-TK, C-CW, H-FC, and C-TL performed the experiments. T-HL, J-TK, D-YL, and C-TL analyzed the data. T-HL, C-CW, and C-TL contributed to reagents, materials, and analysis tools. C-CW and C-TL wrote the manuscript. All authors read and approved the final manuscript.

## Conflict of Interest

The authors declare that the research was conducted in the absence of any commercial or financial relationships that could be construed as a potential conflict of interest.

## Abbreviations

apo-FNR: monomeric FNR
CPS: capsular polysaccharide
DMEM: Dulbecco’s modified Eagle’s medium
ESBL: extended spectrum β-lactamase
FBS: fetal bovine serum
Fur: ferric uptake regulator
holo-FNR: [4Fe-4S] cluster of FNR
KLA: *Klebsiella* liver abscess
LB: Luria-Bertani
ORF: open reading frame
PBS: phosphate-buffered saline.
